# Changes in Youth Cigarette Use Following the Dismantling of an Antitobacco Media Campaign in Florida

**Published:** 2010-04-15

**Authors:** Noella A. Dietz, Lori Westphal, Youjie Huang, Kris L. Arheart, David J. Lee, Evelyn Davila, David F. Sly

**Affiliations:** University of Miami Miller School of Medicine, Sylvester Comprehensive Cancer Center, Department of Epidemiology and Public Health; Florida Department of Health, Tallahassee, Florida; Florida Department of Health, Tallahassee, Florida; University of Miami Miller School of Medicine, Miami, Florida; University of Miami Miller School of Medicine, Miami, Florida; University of Miami Miller School of Medicine, Miami, Florida; Florida State University, Tallahassee, Florida

## Abstract

We examined the association of the termination of a successful youth-targeted antitobacco media campaign ("truth") and changes in smoking rates among youths aged 12-17 years in Florida. Six telephone-based surveys were completed during the active media campaign (1998-2001), and 2 postcampaign surveys were completed in 2004 and 2006 (each n ~1,800). Prevalence of current smoking among youth observed during the campaign continued to decrease in the first postcampaign survey; however, by the second follow-up survey, youth smoking rates had increased significantly for youth aged 16 years or older. Our findings support the need for consistent antitobacco messaging to reduce the prevalence of youth smoking.

## Objective

In 1998, the Florida Department of Health launched the youth-targeted antitobacco "truth" campaign. The Florida Antitobacco Media Evaluation (FAME) surveys were developed to monitor the reach and penetration of the media campaign. A major component of the Florida "truth" campaign was the aggressive antitobacco media campaign. The program had a $78 million budget, anchored by the aggressive media component, which was particularly effective in reducing the prevalence of tobacco use among youth in Florida (1). However, in the 1999-2000 legislative year, funding for the program was reduced to $38.7 million, then reduced to $7.1 million, and in 2003, only $1 million was allocated, essentially eliminating it (2,3).

We examined the effect of terminating a successful youth antitobacco media campaign in Florida. We hypothesized that youth smoking decreased during the life of the antitobacco campaign and increased after the campaign ended.

## Methods

We employed a repeated cross-sectional design. The sampling frames were obtained by vendor-generated lists from which lists of names were randomly selected. Samples were representative of the targeted Florida population by region, sex, ethnicity, and age. Data were collected via telephone interviews, and the sample size for each survey was 1,800 youths, aged 12-17 years. The 6 FAME surveys were timed and designed around the television advertising schedules for the "truth" media campaign and were conducted in September 1998, May 1999, October 1999, May 2000, October 2000, and May 2001. Two postcampaign surveys were conducted in May 2004 and December 2006. The sampling procedures, interview protocols, survey content, and representativeness of the samples have been described previously (4). Participants were asked questions to measure their awareness of the campaign, of specific advertisements, and about their tobacco use. The major outcome items, current smoking status and confirmed advertising or campaign awareness, are measures recommended by the Centers for Disease Control and Prevention for evaluating counter-marketing media programs (5-7).

We report current smoking status for all youth by age from the start of the campaign, September 1998, through the last postcampaign survey, December 2006. Current smoking status was derived from the item, "During the last 30 days, on how many days did you smoke cigarettes, even just a puff or 2?" Responses were coded (smoker = 1; nonsmoker = 0). Distributions of smokers and nonsmokers were compared for successive survey rounds. Respondents were asked, "Are you aware of an antitobacco or antismoking campaign that is now taking place in Florida?" and "What is the theme or slogan of this campaign?" Respondents who answered yes to the first item and then identified the correct theme or slogan were considered to have confirmed campaign awareness (confirm campaign = 1; not confirm campaign = 0).

Advertising awareness also was measured. The first item cued the respondent to the specific television advertisement being asked about: "Have you seen an antismoking advertisement that showed [cue]?" Respondents who gave a positive reply were then asked 2 items to confirm those advertisements with no cues. The first asked the respondent to describe what happened in the advertisement, and the second asked the respondent to describe the main message or theme. Responses were dummy coded (confirm advertisement/not confirm advertisement). Because direction of change is predicted, 1-tailed χ^2^ tests were employed to determine significance. This study was approved by the University of Miami Human Subjects Committee; participants gave informed consent or assent.

## Results

By the end of the first year of the "truth" campaign, 96% of youth confirmed they were aware of the "truth" campaign, and 93% of youth confirmed awareness of at least 1 "truth" advertisement (4). Confirmed advertising awareness remained high throughout the campaign. The rate of current smoking for all youth declined from baseline to campaign termination in 2001 by 31.3% [χ^2^(1, N = 1,800) = 18.26, *P* = .001] (Figure). For youth aged 16 years or older, cigarette use decreased by 34.9% from baseline to campaign termination χ^2^(1,1, N = 1,800) = 19.80, *P* = .001] and by 18.6% for youth aged 15 years or younger [χ^2^(1,1, N = 1,800) = 2.88, *P* = .05].

**Figure.**
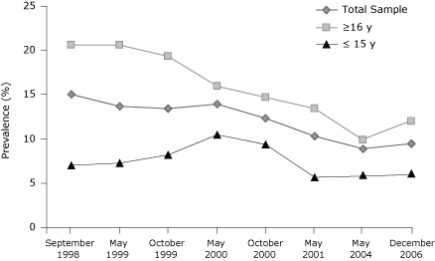
Prevalence of smoking among Florida youth aged ≤15 years, aged ≥16 years, and the entire sample (N = 1,800), September 1998-December 2006. Data for 1998-2001 were collected during the "truth" antitobacco media campaign; data for 2004 and 2006 were collected after the campaign ended.

**Table:** 

**Population**	**Prevalence (%)**							

	**Sept. 1998**	**May 1999**	**Oct. 1999**	**May 2000**	**Oct. 2000**	**May 2001**	**May 2004**	**Dec. 2006**
≤15 y	7.0	7.3	8.2	10.5	9.4	5.7	5.9	6.1
≥16 y	20.6	20.6	19.3	16.0	14.7	13.4	9.9	12.0
Total Sample	15.0	13.7	13.4	13.9	12.3	10.3	8.9	9.5

The data for the 2 postcampaign survey intervals (2001-2004 and 2004-2006) allowed us to assess the effects of termination. During the first interval, smoking continued to decline for all youth after the campaign ended (14.6%) [χ^2^(1, N = 1,800) = 2.99, *P* = .05], but at a slower pace. This finding also was true for youth aged 16 years or older (26.1%) [χ^2^(1, N = 1,800) = 6.97, *P* = .001]. For youth aged 15 years or younger, smoking increased slightly but not significantly. During this period, most youth were still able to confirm awareness of the advertising campaign (64.2%), indicating that they recalled advertisements that had not run in 4 years. However, for the second postcampaign interval (2004-2006), smoking rate declines started to reverse for youth aged 16 years or older. For all youth, the rate of smoking increased by 6.8%, and for youth aged 15 years or younger, rates increased by 11.9% (neither significant). However, for youth aged 16 years or older, the rate of smoking increased by 21.2% [χ^2^(1, N = 1,800) = 2.59, *P* = .05]. We also saw that the number of youth who were able to confirm awareness of the advertising declined (10.5%).

## Discussion

The "truth" antitobacco media campaign targeted youth to prevent them from smoking. This study differs from previous reports (2,8) because it focuses on a longer-term impact of campaign termination and uses current cigarette use as the primary outcome measure. Our hypothesis predicted that smoking would decrease during the campaign and increase after the campaign ended. Three years after termination, smoking continued to decline, which may have reflected continued campaign effects and possibly effects on younger youth who remained in the targeted ages after the campaign ended. The significant increase in current smoking was observed only after all youth in the original study cohorts had reached young adulthood and were no longer captured in our youth surveys. These young adults who were exposed to the "truth" campaign as youths continued to be less likely to report smoking (9). These smoking trend patterns also are reflected in data from the Florida Youth Tobacco Survey. However, the FAME samples capture all people in the targeted ages, not just samples of youth attending school (10).

There were limitations to this study. First, since the design was cross-sectional, individual changes in smoking behavior could not be determined. However, repeated cross-sectional surveys can track youth campaign and advertising awareness. Also, because our samples were not true population-based estimates, some caution should be used when generalizing these results to the Florida population.

Cigarette use continued to decline immediately after the program ended, which possibly reflected the campaign's continued inhibiting effect on cigarette uptake among youth who were youngest when the campaign was operative and aged into the older youth ages after campaign termination. Our findings suggest that within a 5-year period, the positive effects of this successful youth-targeted antitobacco media campaign were clearly halted and are potentially reversed. When the youth population became composed entirely of people with limited campaign exposure, population-level rates of youth smoking stabilized and began to increase. These results support the need for continuous and adequate funding of antitobacco media campaigns targeted at youth as part of a comprehensive state tobacco control effort.
